# Simulation-assisted multimodal deep learning (Sim-MDL) fusion models for the evaluation of thermal barrier coatings using infrared thermography and Terahertz imaging

**DOI:** 10.1038/s41598-025-31783-8

**Published:** 2025-12-08

**Authors:** Sruthi Krishna Kunji Purayil, Sachinlal Aroliveetil, Adarsh Chaturvedi, Krishnan Balasubramaniam

**Affiliations:** 1 Thermographic Methods, Federal Institute for Materials Research and Testing (BAM), 12205 Berlin, Germany; 2 Center for Non-Destructive Evaluation, Indian Institute of Technology Madras, Chennai, 600036 India

**Keywords:** Multimodal fusion, Deep learning, Thermal barrier coatings (TBC), Infrared thermography, Terahertz, Thickness, Non-destructive evaluation (NDE), Energy science and technology, Engineering, Materials science, Mathematics and computing

## Abstract

Thermal Barrier Coatings (TBCs) are critical for high-temperature applications, such as gas turbines and aerospace engines, protecting metallic substrates from extreme thermal stress and degradation. Accurate evaluation of TBCs is essential to improve operational efficiency, optimize predictive maintenance strategies, and extend component life. Conventional non-destructive evaluation (NDE) techniques such as infrared thermography (IRT) and terahertz (THz) imaging have been widely used for TBC inspection with limitations when used independently, including sensitivity to surface conditions, limited penetration depth mainly in multi-layer coatings. This study proposes a novel framework called simulation-assisted multimodal deep learning (Sim-MDL) that combines IRT and THz data for a comprehensive evaluation of TBCs. To generalize the study to varying thermophysical properties of TBCs, the study uses simulation-generated data along with experimental data for training deep learning models. Two deep learning frameworks based on a 1D convolutional neural networks (CNN) and a long short-term memory (LSTM) with attention were developed for the multimodal feature fusion. The IR-THz fused frameworks enable simultaneous prediction of key TBC topcoat properties including thermal conductivity, heat capacity, topcoat thickness and refractive index. Experiments were conducted on four newly coated samples topcoat thicknesses ranging from 24 to 120 μm. An attention-based LSTM model trained on both simulation and experimental data shows high prediction accuracy with MAPE values ranging from 2.06% to 4.43% for thermal conductivity, 2.05% to 3.57% for heat capacity, 11.53% to 1.75% for topcoat thickness, and 0.27% to 1.05% for refractive index, respectively, for the topcoat layers of four samples. The proposed Sim-MDL framework outperformed single-modality and conventional parameter estimation methods in accuracy and robustness, highlighting the potential of multimodal data for automated analysis of TBC in industrial settings.

## Introduction

TBC are crucial in high-temperature applications, including gas turbines and aircraft engines, due to their thermal insulation and protects components from severe thermal stresses^1^. Extensive study has been undertaken to comprehend the deterioration mechanisms of TBC and to develop advanced NDE techniques for assessing their distinctive features and lifespan^2–4^. This section examines recent research on TBC evaluation, emphasizing current progress in NDE techniques, the integration of simulation-assisted methods, and the application of deep learning frameworks^5^.

Standard NDE techniques, such as IRT and THz imaging, have been commonly used for the evaluation of TBC^6^. IRT has been shown to be effective at detecting surface and subsurface defects in TBCs by analyzing thermal responses under transient heating conditions^7,8^. Researchers have shown that thermal imaging is effective in detecting delamination, cracks, and differences in thermal conductivity^9–12^. Nonetheless, limitations such as sensitivity to surface emissivity and lower penetration depth have limited its use in multilayer coatings and subsurface characterization^13,14^. THz imaging has improved penetrating capabilities, making it useful for evaluating multilayer structures and detecting subsurface defects^15–17^. Research has demonstrated its capability to estimate topcoat layer thickness measurements, identifying delamination, and predicting refractive index^18–20^. Despite its benefits, THz imaging is limited by noise sensitivity and poor spatial resolution, which might reduce its efficacy when used as a single inspection modality^21,22^.

Simulation approaches have become essential tools for producing synthetic datasets and comprehending the thermophysical properties of TBCs^23^. Finite-element models are extensively utilized to estimate heat reactions and degradation mechanisms in TBCs^24,25^. These models facilitate the prediction of thermal conductivity, heat capacity, and variations in thickness during the service life^26^. Data generated through simulation is widely utilized to supplement restricted experimental datasets, hence improving the training and validation of prediction models. Recent studies have shown the effectiveness of integrating simulation and experimental data for property prediction and degradation analysis^27–29^.

The incorporation of ML and DL methodologies into NDE has transformed the assessment of TBCs by facilitating automated and precise property prediction^30,31^. Unimodal AI models developed based on machine learning methods, such SVM and ANN, to correlate NDE data with TBC features^32–34^. Deep learning algorithms utilizing CNNs to analyze microscopic cross-section images of TBCs, showcasing enhanced precision in forecasting thermal and structural deterioration^35^. A deep learning framework based on CNNs developed to predict the coatings failure. This prediction is done by integrating multi-scale imaging data and used efficiency of fusing complementing modalities for improved assessment^36^. Despite the advancements, several challenges in TBC evaluation:

- Unavailability of experiment data for training the data-driven AI models.
- Challenges in generalizing models across varied material compositions, coating thicknesses, and operational conditions.
- Challenges in correlating multimodal NDE data due to inconsistencies in spatial and temporal resolution across different test methods.

Although prior research has substantially advanced TBC assessment, most of them concentrated on single NDE modalities or conventional machine learning frameworks for the evaluation TBC performance^37–39^. The integration of IRT and THz will be a practical solution to overcome redundancy in a single modality and provides complementary information^40,41^. The promise of multimodal data fusion and sophisticated deep learning architectures, including attention-based models, is yet inadequately investigated^42–46^. Recent studies have explored multimodal fusion in material evaluation and defect detection based on IR and THz imaging. However, most of these works dependent solely on the experiment data and not taken advantage of the physics-based simulation data to improve the generalization of the prediction model.

This paper proposes a unique simulation-assisted multimodal deep learning methodology that evaluates TBC properties, by solving the observed limitations. The proposed framework combines IRT and THz test data with synthetically generated dataset, overcoming the limitations of single-modal NDT evaluation method and thereby improving the model generalization. The application of advanced architectures, such as LSTM with attention mechanisms, allows for accurate prediction of thermophysical properties, demonstrating a significant advancement in the automated evaluation of TBC coatings.

## Data acquisition

### Sample Preparation

**Fig. 1:**
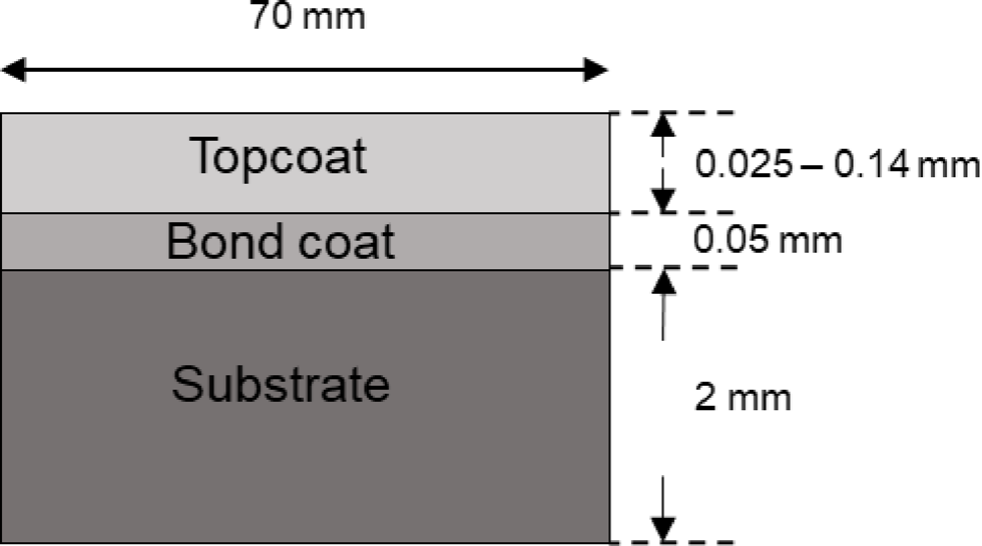
Schematic cross-section of the TBC sample used in this study.

**Fig. 2:**
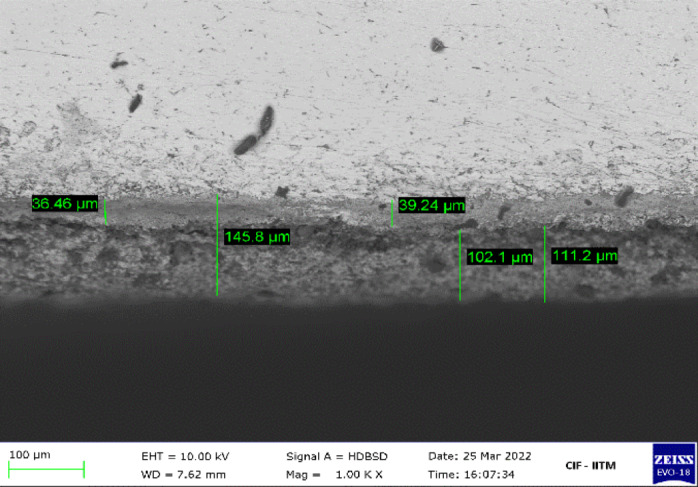
SEM image of cross-sectional microstructures of TBC: sample with mean topcoat thickness of 100 μm and bond coat thickness of 50 μm.

The pulsed thermal imaging experiment setup and sample configuration used in this study closely follow our previous work which laid the foundation for the simulation-assisted AI model for the evaluation of TBC using thermal NDE data^27^. In this study, four TBC coupon specimens are examined using both pulsed thermal imaging and terahertz imaging experiments. The schematic of the cross-section of the newly coated TBC sample used in the study is shown in Fig. 1. Each sample consists of three layers: a nickel-based superalloy substrate, a NiCoCrAlY bond coat deposited via atmospheric plasma spraying (APS), and a topcoat of yttria-stabilized zirconia (YSZ). All four specimens have surface dimensions of 70 mm x 70 mm each, bond coat layer of thickness 50 μm, and a topcoat thickness ranging from 24 to 120 μm. SEM is performed to measure the layer thicknesses of samples. SEM image of the cross-sectional microstructure of TBC for a sample with a mean topcoat and bond coat layer thickness of 100 μm and 50 μm, respectively, is shown in Fig. 2. To minimize surface reflectivity and optical translucency of the ceramic topcoat, a thin layer of graphite-based black paint was applied on the topcoat surface^27–29^.

### Pulsed infrared thermography

#### Experiment

**Fig. 3:**
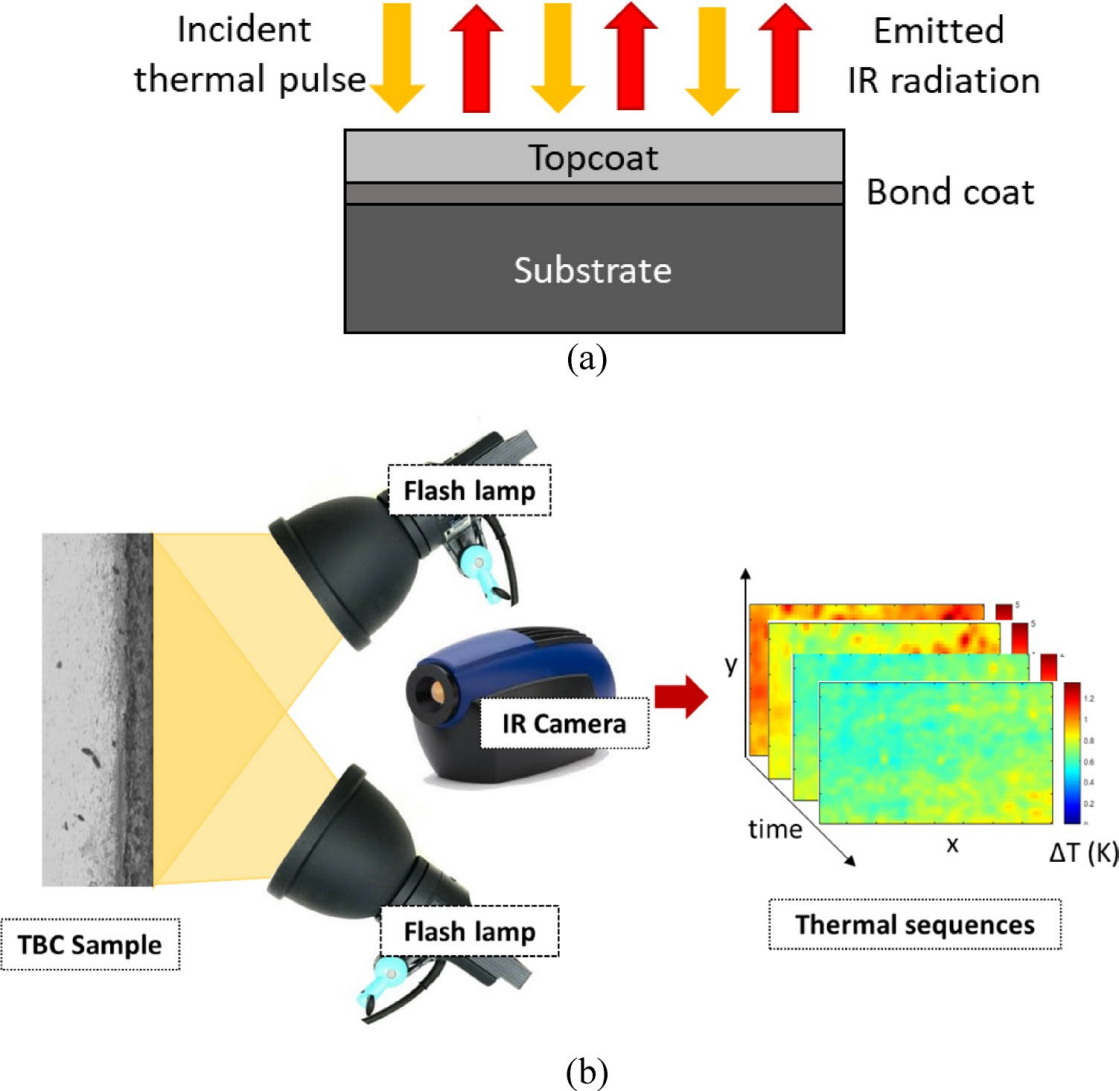
(**a**) Schematic of infrared thermography on TBC sample with topcoat, bond coat and substrate, (**b**) Schematic of the infrared thermal imaging experiment setup.

In active infrared thermography, a short duration photothermal pulse is applied to the specimen surface to induce a temperature difference and observe the temperature rise using an infrared camera. In the Fig. 3(a)., shows the schematic diagram of basic IR inspection on TBC used in this study. Incident excitation is representation of applied short-duration heat pulse and emitted IR radiation from the sample surface. In the Fig. 3(b), shows the experiment setup used for the testing of TBC sample. Two flash lamps with 400 J of energy for a time duration of 1.1 ms is used as active heat source to excite the TBC sample surface. A high-resolution mid-wavelength infrared (MWIR) camera of wavelength 3–5 μm is used to record the heat transport through the specimen with a frame rate of 3 kHz.

#### Heat transfer model

The numerical simulations of heat transfer model for pulsed thermal imaging were adopted from our earlier study and extended to the current study combined with terahertz imaging^27^. These heat transfer models are used to study the influence of topcoat thickness on transient temperature responses. All three layers of the specimen are modelled as isotropic and homogeneous. The initial and ambient temperatures are both set at 300 K. The surface of the specimen is excited with a short pulse of 1.1 ms pulse duration with an energy of 1.6 kJ. Due to the low thermal conductivity of the ceramic topcoat compared to the metallic layers, the heat diffusion is relatively slow through the specimen. Since, the incident heat pulse is uniform, the transient heat diffusion occurs primarily in the depth direction of the sample. Considering the geometry of the specimen, the 3D heat transfer model can be approximated to 1D, which significantly reduced the computational time from ~ 25 h to ~ 60 s. This reduced computational cost enabled large-scale dataset generation for training the simulation-assisted deep learning models. The simulation dataset (3000 nos) was generated varying topcoat thermal conductivity, and heat capacity values by varying ± 5% from their reference values and by varying topcoat thickness from 20 $\:\mu\:m$ to 200 $\:\mu\:m$.

The governing one-dimensional transient heat-conduction equation is given by

1 $$\:\rho\:{C}_{p}\frac{\partial\:T}{\partial\:t}=\:\frac{\partial\:}{\partial\:x}\left(k\frac{\partial\:T}{\partial\:x}\right)\:+Q\left(x,t\right)$$

with the boundary condition

2 $$\:-k\frac{\partial\:T}{\partial\:x}=h\left(T-{T}_{\infty\:}\right)+\:ϵ\sigma\:\left({T}^{4}-\:{T}_{\infty\:}^{4}\right)$$

Where $\:\rho\:$, $\:{C}_{p}$, and $\:k$ are the density, specific heat capacity, and thermal conductivity of layer, respectively, and $\:Q(x,t)$ represent the volumetric heat source generated by the photothermal excitation. In Eq. (2), $\:h$ and $\:ϵ\sigma\:$ denotes the convective and heat-loss terms respectively. The initial and boundary conditions at surfaces are considered as adiabatic^28,29^.

### Terahertz imaging

#### Terahertz imaging experiment

**Fig. 4:**
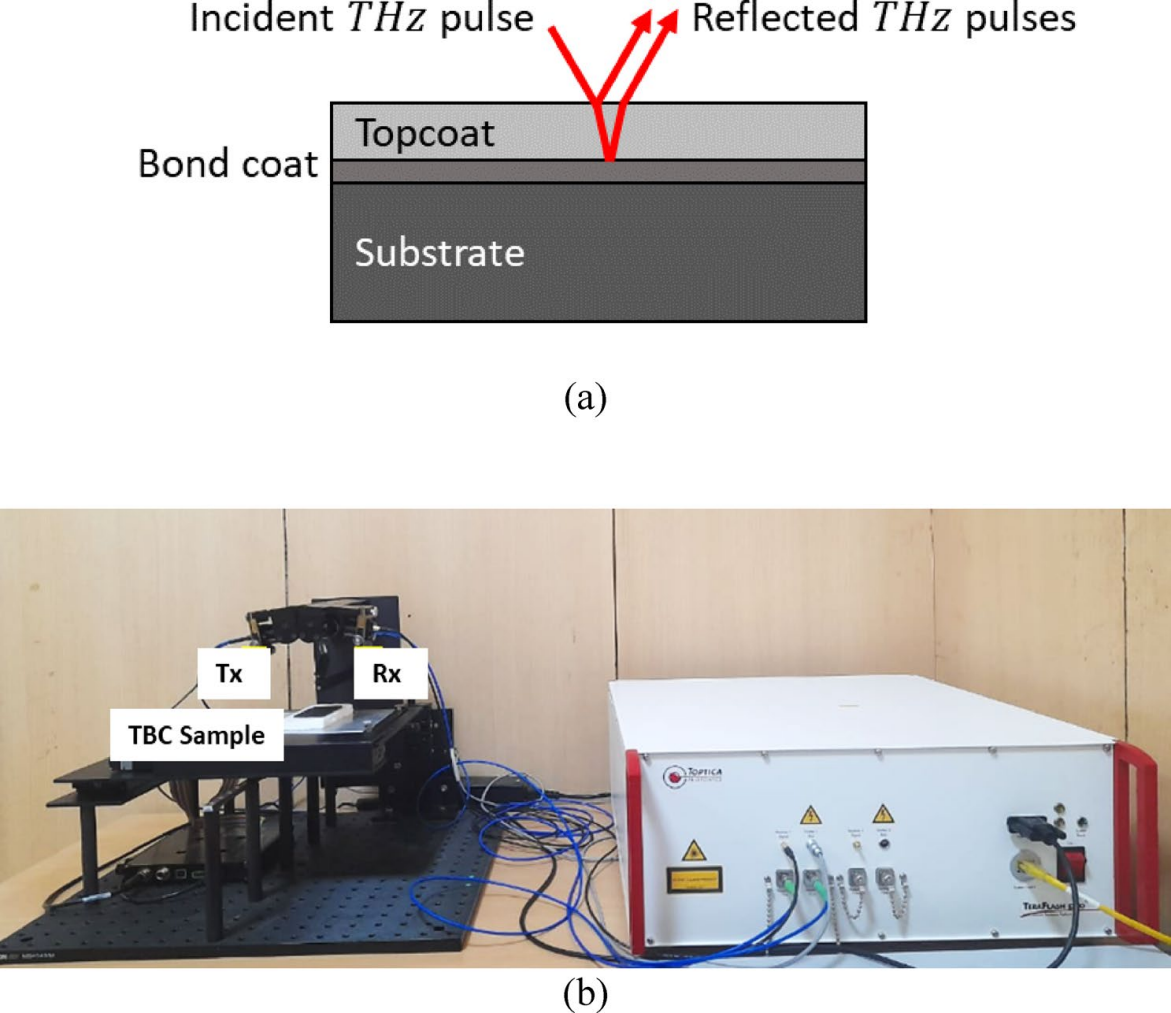
(**a**) Schematic of a THz imaging on TBC sample with topcoat, bond coat and substrate, (**b**) Experiment setup of THz imaging of TBC sample showing transmitter (Tx) and receiver (Rx).

THz spectroscopy is an NDE method that uses electromagnetic waves in the terahertz frequency range (0.1–10 THz). This method is particularly effective for evaluating multilayered materials, such as TBC, due to its ability to penetrate non-metallic materials and provide information about internal structures and material properties^47^. A terahertz beam is directed at the TBC sample surface. The incident THz pulse interacts with the bond coat, resulting in reflection and transmission. The reflected THz pulses from the interface between topcoat and bond coat contain information about topcoat layer thickness and refractive index. Changes in the wave’s phase and amplitude provide insights into material properties, such as the n and $\:{T}_{topcoat}$. The schematic of the THz imaging of TBC coating is shown in Fig. 4 (a) and experiment setup used for the study is shown in Fig. 4(b). The reference reflected pulse from the topcoat surface and reflected pulse from the interface between topcoat and bond coat is recorded using receiver. A terahertz detector records the reflected and transmitted signals, which are processed to extract features relevant to the TBC layers. The time-of-flight difference between the reference reflected pulse and reflected pulse from interface is the basis for the measurement of topcoat thickness.

#### Terahertz simulation

**Fig. 5:**
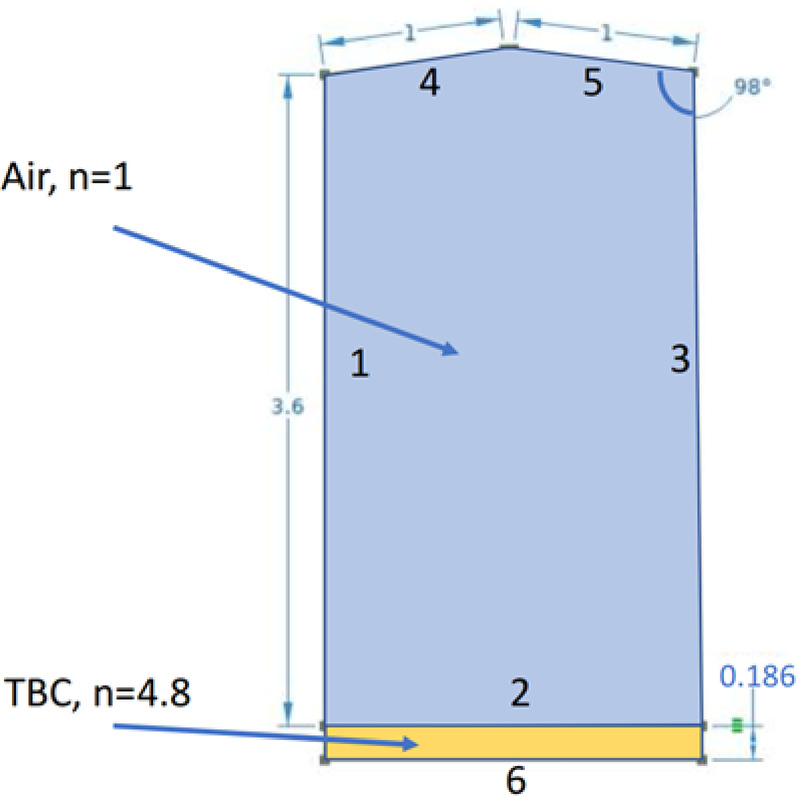
Schematic of a 2D-numerical model of TBC with the topcoat layer thickness, $\:{T}_{topcoat}=\:186\:{\upmu\:}\text{m}$, and refractive index, $\:n=4.8$

The simulation is used to generate synthetic data that complement experimental measurements. The COMSOL Multiphysics is used for simulating terahertz wave propagation through multilayer structures. The simulation is used to generate synthetic data that complement experimental measurements. A 2D simulation domain has been formulated to simulate the reflection of THz pulses from the topcoat layer. Since the bond coat is metallic in nature, THz pulses will be completely reflected from the topcoat-bond coat interface. To account for this, a perfect electrical conductor (PEC) boundary condition has been applied to this interface. This mimics the entire metallic reflection of the THz pulses in the TBC multi-layered coatings. The simulation domain represents the multilayer TBC system, consisting of the topcoat layer and the bond coat layer, as shown in Fig. 5. The topcoat thickness measurement ranges of samples range from 24 μm to 120 μm. Scattering boundary conditions with absorbing constraints are applied to the domain boundaries to prevent artificial reflections from interfering with the simulation. A user defined mesh with an element size of (1/12)^th^ of the wavelength (maximum frequency was 1THz) was chosen to discretize the topcoat layer. The reference value for mean refractive index, $\:n\:\approx\:4.8$ for APS-YSZ in the terahertz band was adopted based on the previous THz-TDS studies^39,48^.

The excitation pulse for the simulation is extracted from a reference signal collected at the receiver (Rx) after placing an aluminum plate as a perfect reflector in THz beam path. The electromagnetic waves (time domain) ewt module is used to solve Maxwell’s equations for terahertz wave propagation in COMSOL. The interaction of the terahertz wave with the topcoat and substrate is simulated to generate reflected and transmitted signals. The 2D simulation is used to generate synthetic dataset (3000 nos) by varying topcoat thickness from $\:20\:\mu\:m$ to $\:200\:\mu\:m$ and $\:n\:=\:4.8\pm\:5\:\%,$ such as the phase shift and amplitude attenuation of terahertz waves at the interfaces. These results are used to: validate experimental measurements, train machine learning models, such as Sim-MDL, by creating large datasets representing various material properties and conditions.

## Simulation-assisted multimodal deep learning (Sim-MDL) framework

In this study two Multimodal DL models are designed and optimized based on DL architectures such as CNN and LSTM. Late-stage fusion is done on features extracted from two models given input of IR and THz signal separately and in the final stage, thermal properties of TBCs are predicted using a fully connected neural network. Two Sim-MDL frameworks based on 1D-CNN and LSTM + Attention based networks are explained in the subsections.

### D - Convolution neural network (CNN) based Sim-MDL

**Fig. 6:**
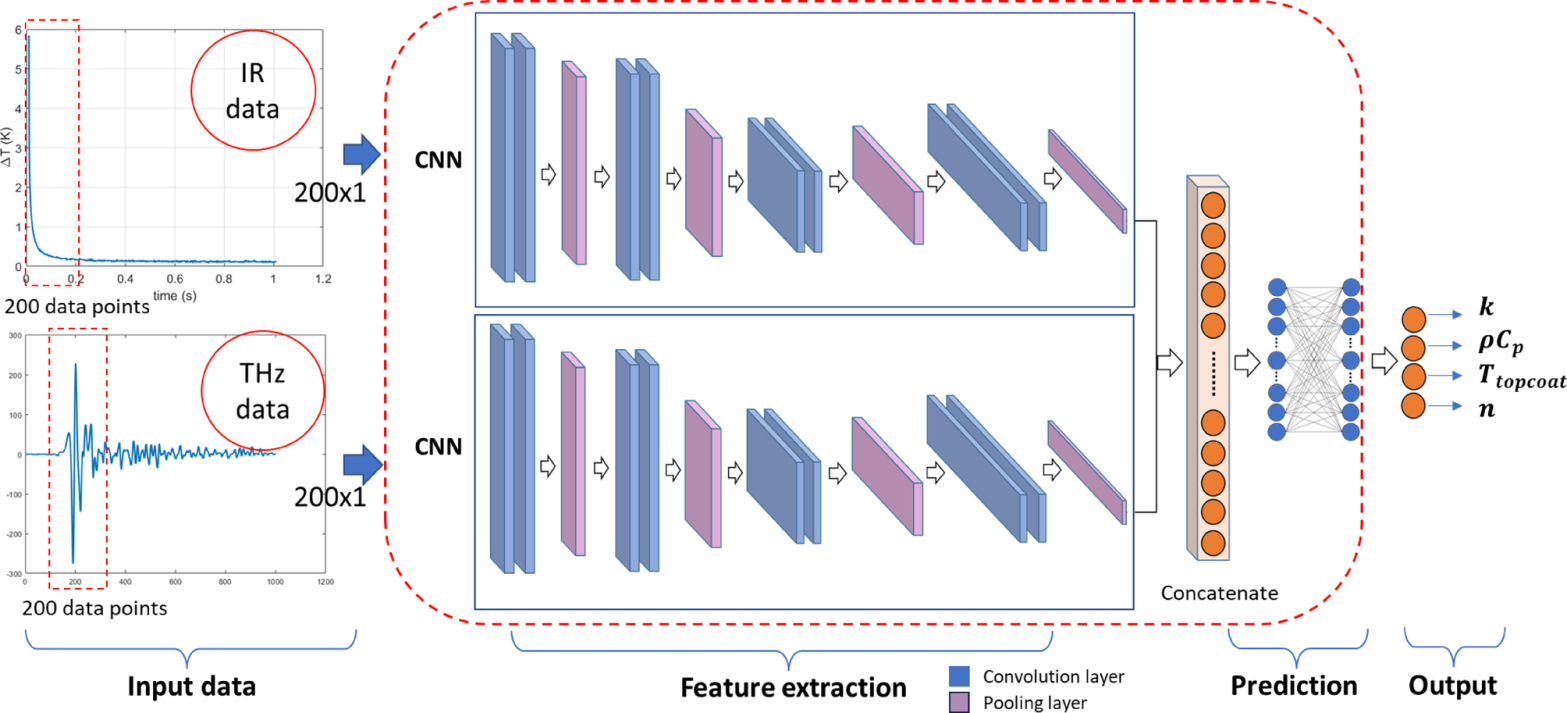
Schematic of the 1D-CNN based framework for the prediction of TBC properties by fusing features from IR and THz data.

The first framework utilizes a CNN architecture designed to handle multimodal data-specifically IR data and THz data for the prediction of TBC properties. The model is structured to process two types of data using separate models and fuse the features in in the later stage to predict $\:k$, $\:\rho\:{C}_{p}$​, $\:{T}_{topcoat}$ and $\:n$. Figure 6. shows the schematic of the 1D-CNN based framework for the prediction of TBC properties by fusing features from IR and THz data. The input data given to the first model is a vector of 200 temperature values over time from thermal imaging and to the second model is a vector of 200 spectral data points from THz imaging. Each dataset is processed through CNN model separately and features are extracted using convolution and pooling layers.

3 $$\:{f}_{i}=ReLU\:({W}_{i}*x+\:{b}_{i})$$

4 $$\:{p}_{i}=MaxPool\left({f}_{i}\right)$$

The feature map of $\:{i}^{th}$layer $\:{f}_{i}$ is given in the Eq. (3), Wi is the weight of $\:{i}^{th}$ layer, $\:{b}_{i}$ is the bias, $\:x$ is the input feature map and $\:ReLU$ is the rectified linear unit. A pooling layer is used to reduce the size of the features from convolution layers and to decrease the computation. Where $\:{p}_{i}$ is the output of pooling operation on feature map $\:{f}_{i}$. After extracting features from both datasets, outputs from each CNN blocks are concatenated into a single vector, F = [$\:{f}_{IR};{f}_{THz}$]. The concatenated vector is then fed into fully connected layers, which gives the final output predictions to the output layer.

### Long short-term memory (LSTM) with attention-based Sim-MDL

**Fig. 7:**
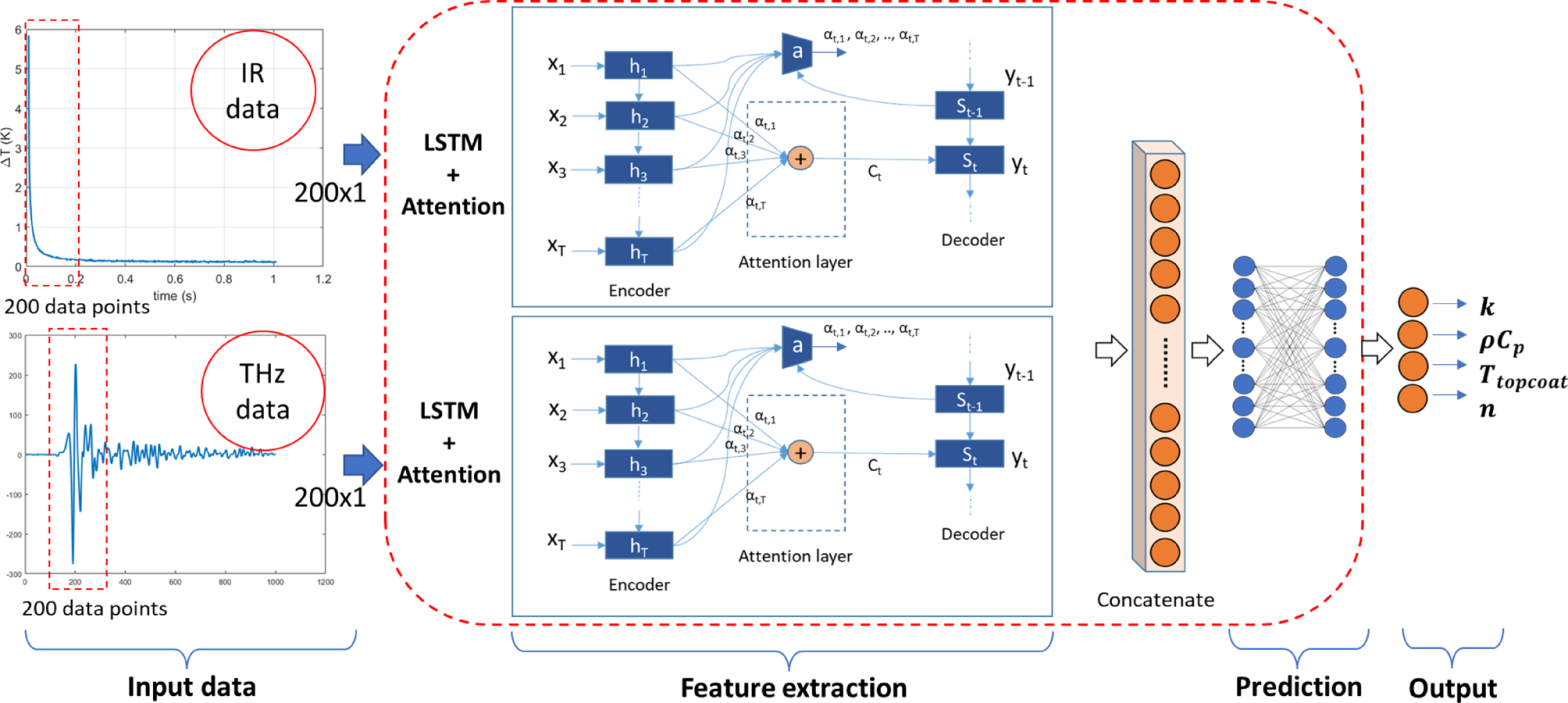
Schematic of the LSTM + Attention based framework for the prediction of TBC properties by fusing features from IR and THz data.

The second framework describe the LSTM network with attention mechanism modelled to process and fuse features of multimodal data inputs. Similar to the CNN framework, this model inputs IRT and THz data as input and predict TBC properties such as $\:k$, $\:\rho\:{C}_{p}$, $\:{T}_{topcoat}$ and n. However, instead of focusing on convolutional feature extraction, the LSTM-based framework captures the temporal dependencies and sequential patterns in both data modalities. Figure 7. shows the schematic of the LSTM + Attention based framework for the prediction of TBC properties by fusing features from IR and THz data. The input data given to the first model is a vector of 200 temperature values over time from thermal imaging and to the second model is a vector of 200 spectral data points from THz imaging. Each dataset is processed through LSTM model separately and features are extracted.

5 $$\:{i}_{t}=\:\sigma\:({W}_{i}\cdot\:\left[{h}_{t-1},\:{x}_{t}\right]+{b}_{i})$$

6 $$\:{f}_{t}=\:\sigma\:({W}_{f}\cdot\:\left[{h}_{t-1},\:{x}_{t}\right]+{b}_{f})$$

7 $$\:{o}_{t}=\:\sigma\:({W}_{o}\cdot\:\left[{h}_{t-1},\:{x}_{t}\right]+{b}_{o})$$

8 $$\:{g}_{t}=\:tanh({W}_{c}\cdot\:\left[{h}_{t-1},\:{x}_{t}\right]+{b}_{c})$$

9 $$\:{c}_{t}=\:{f}_{t}\odot\:{c}_{t-1}+\:{i}_{t}\odot\:{g}_{t}$$

10 $$\:{h}_{t}=\:{o}_{t}\odot\:{\text{t}\text{a}\text{n}\text{h}(c}_{t})$$

Where $\:{i}_{t}$, $\:{f}_{t}$ and $\:{o}_{t}$ represents the input gate, forget gate and output gates, $\:{g}_{t}$is the cell activation vector, $\:{c}_{t}$is the cell state, $\:{h}_{t}$is the hidden state, $\:{x}_{t}$ is the input at time $\:t$, and $\:\sigma\:$ is the sigmoid activation function used in the LSTM unit. Output from LSTM layer is passed through attention layer that assigns dynamic weights to the different timesteps based on hidden states.

11 $$\:{c}_{t}=\:\sum\:_{t=1}^{T}{\alpha\:}_{t}{h}_{t}$$

Where $\:{\alpha\:}_{t}$ are the attention weights and $\:{c}_{t}$ is the context vector. The context vector from both IR data and THz data is concatenated to form a single vector, Y = [$\:{Y}_{IR};{Y}_{THz}$]. The concatenated vector is then fed into fully connected layers, which gives the final output predictions to the output layer.

### Training procedure and optimizing techniques

#### CNN-Based multimodal framework

The input data consists of IRT (temperature decay profiles) and THz (spectral information) measurements, each consisting of 200 data points. These datasets are pre-processed to normalize the inputs and ensure consistency across samples. Simulation-generated data is augmented with experimental data to expand the dataset and improve model generalization. Each input stream (IRT and THz) is fed into separate CNN branch, where convolutional layers extract hierarchical features from the input data and pooling layers reduce the spatial dimensions while retaining critical information, helping the model focus on essential patterns. After feature extraction, features vectors obtained from the IR and THz branches (each of 10,000 features) are concatenated to form a single vector of 20,000 features. The concatenated features are passed through fully connected dense layers, which output predictions for multiple properties: $\:k$, $\:\rho\:{C}_{p}$, $\:{T}_{topcoat}$ and $\:n$. This late-stage fusion integrates complementary information from the two modalities, allowing the model to leverage the strengths of both IRT and THz data.

The model is trained using backpropagation with the Adam optimizer, which adjusts the model parameters to minimize the combined loss across all predicted properties. The training is conducted over multiple epochs, with mini batches to improve convergence. A dynamic learning rate is employed to start with a higher value for faster convergence, which is gradually reduced to fine-tune the weights. Dropout is used in the fully connected layers to prevent overfitting by randomly deactivating neurons during training. Training stops automatically if the validation loss stops improving after a predefined number of epochs to prevent overfitting. A loss function, typically the MAPE, is calculated for each property to measure prediction accuracy.

#### Attention-based LSTM framework

IRT and THz data are normalized and aligned as sequential inputs similar to the CNN framework. Simulation data complements the experimental dataset, ensuring a wide range of scenarios for training. Each modality (IRT and THz) is fed into separate LSTM branch. The LSTMs capture temporal dependencies and sequential patterns, such as temperature decay trends in IRT or spectral peaks in THz data. An attention layer is applied to the LSTM outputs, as this mechanism assigns weights to specific time steps, enabling the model to focus on the most relevant temporal features. For example, key thermal decay points in IRT or significant spectral features in THz imaging are given more importance. After feature extraction, features vectors obtained from IR and THz branches (each of 10,000 features) are concatenated to form a single vector of 20,000 features. The concatenated features are passed through fully connected dense layers, which output predictions for multiple properties: $\:k$, $\:\rho\:{C}_{p}$, $\:{T}_{topcoat}$ and $\:n$. This late-stage fusion integrates complementary information from the two modalities, allowing the model to leverage the strengths of both IRT and THz data.

The loss is computed using MAPE for each property, and the total loss is minimized during training. The model is trained using backpropagation, with the Adam optimizer and mini batches to optimize the weights of both the LSTM and attention layers. Training is performed over multiple epochs, with both training and validation datasets used to monitor performance. To prevent the attention mechanism from overfitting to noise, regularization techniques such as penalizing overly sharp attention distributions are employed. LSTMs are prone to exploding gradients; hence, gradient clipping is applied to constrain the magnitude of gradients during backpropagation. Applied to stabilize and accelerate training by normalizing layer outputs.

## Results and discussion

The Sim-MDL models are trained, tested, and validated with data from IR imaging and THz imaging of TBC samples. The provided results illustrate the performance of two deep learning models, the 1D-CNN, and the LSTM + Attention, across two different training scenarios: models trained with only simulation data and models trained with combination of simulation and experimental data. These results assess effectiveness of models in predicting $\:k$, $\:\rho\:{C}_{p}$, $\:{T}_{topcoat}$, and $\:n$ for TBCs.

### Prediction results of models trained with simulation data

**Fig. 8:**
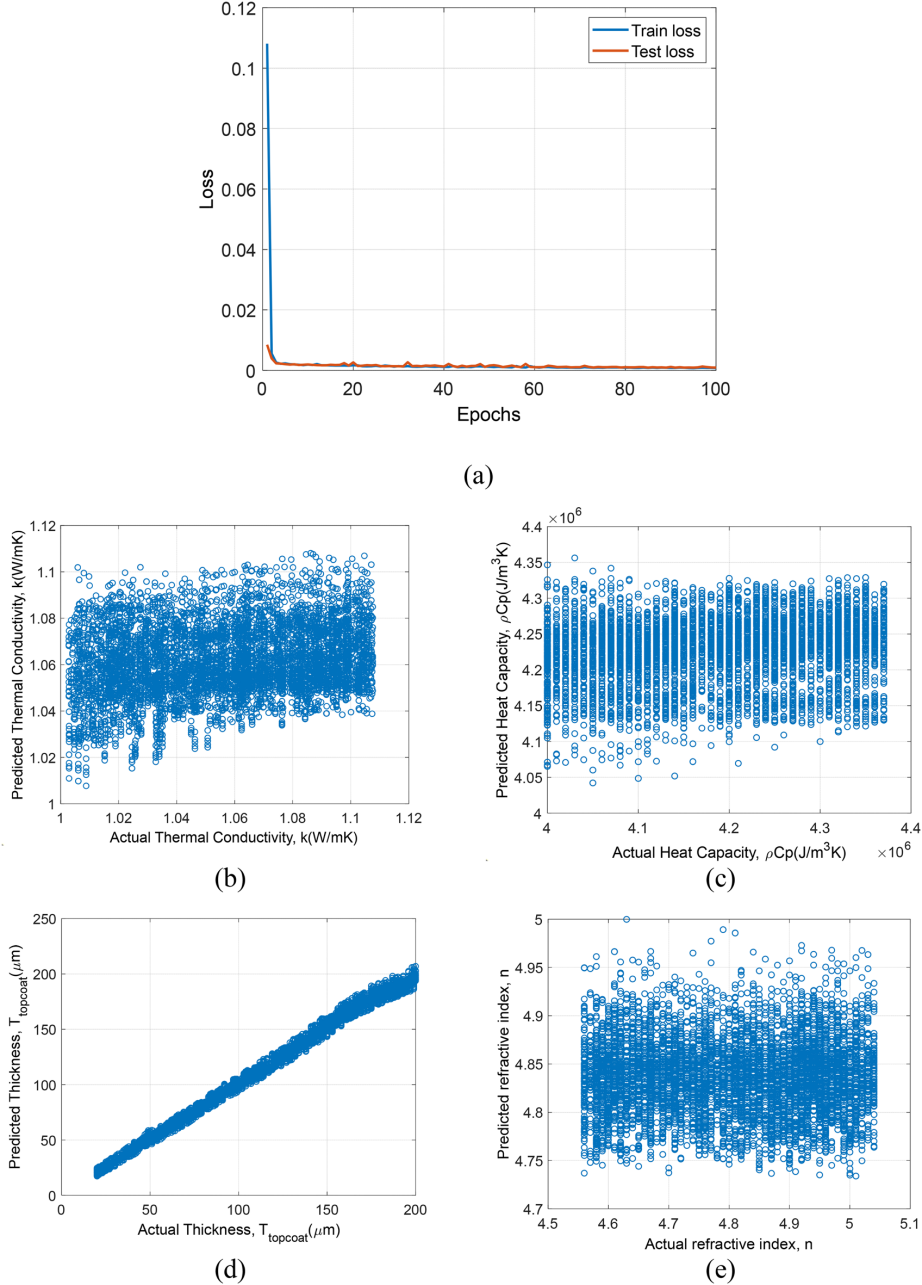
Prediction results for 1D-CNN framework trained and tested on simulation data: (**a**) epochs vs. loss, (**b**) Actual $\:k$ vs. predicted $\:k$ values, (**c**) actual $\:\rho\:{C}_{p}$ vs. predicted $\:\rho\:{C}_{p}$, (d) actual $\:{T}_{topcoat}$ vs. predicted $\:{T}_{topcoat}$, and (**e**) actual $\:n$ vs. predicted $\:n$.

In the first section, we discuss models which are trained and tested with only simulation data. In Fig. 8., the prediction results of model based on 1D-CNN trained with only simulation data is plotted. In Fig. 8(a), epochs vs. loss for train and test set is shown. The model shows quick convergence within the first 20 epochs, indicating an effective learning rate and model architecture for the simulated dataset. In Fig. 8(b), 8(c), 8(d) and 8(e) shows the prediction results of $\:k$, $\:\rho\:{C}_{p}$, $\:{T}_{topcoat}$ and n respectively, for test dataset. Predictions for k and $\:\rho\:{C}_{p}$ show considerable scatter, suggesting variability in model performance. Predictions for $\:{T}_{topcoat}$ and $\:n$ are more accurate, with closer alignment to the actual values.

**Fig. 9:**
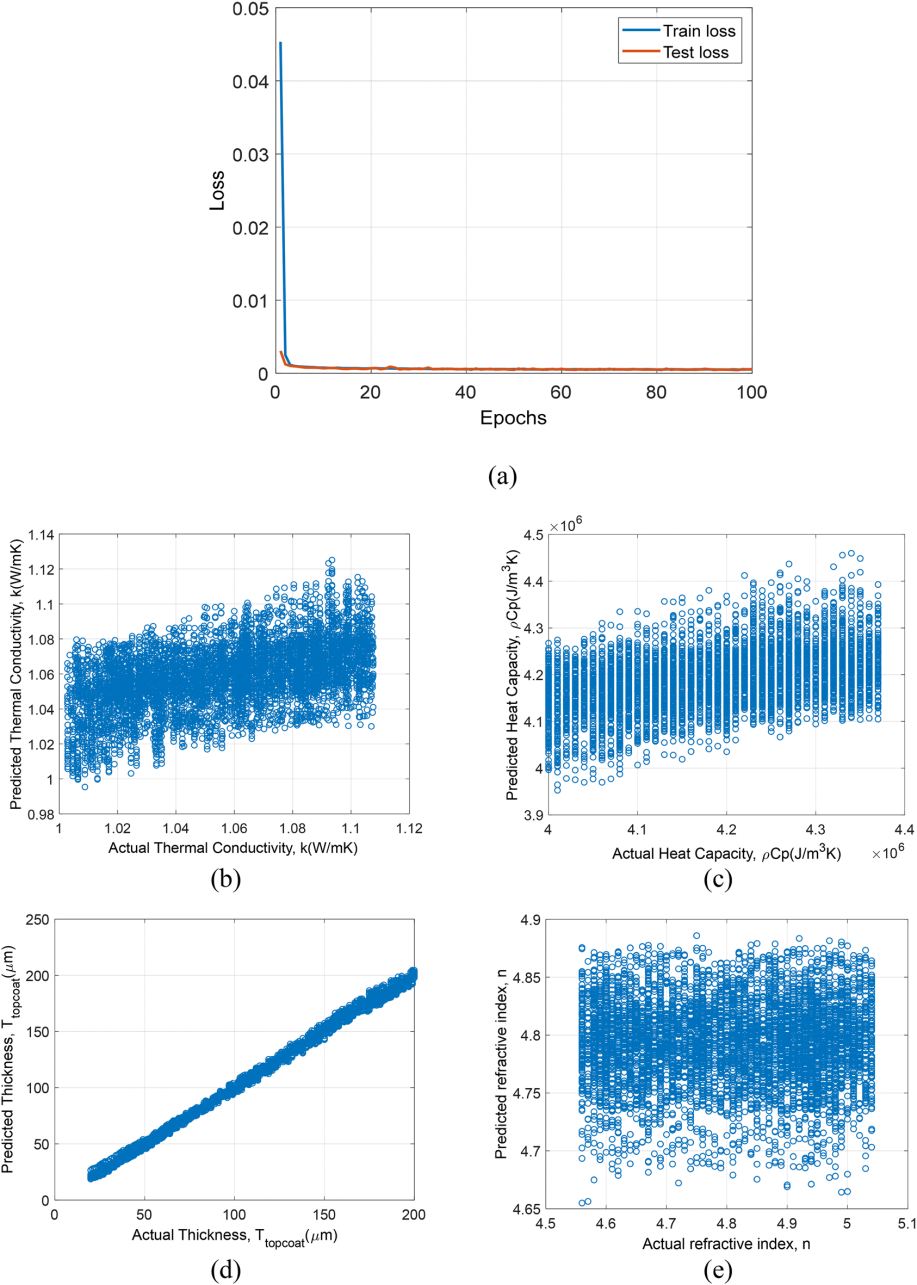
Prediction results for LSTM + Attention framework trained and tested on simulation data: (**a**) epochs vs. loss, (**b**) Actual $\:k$ vs. predicted $\:k$ values, (c) actual $\:\rho\:{C}_{p}$ vs. predicted $\:\rho\:{C}_{p}$, (**d**) actual $\:{T}_{topcoat}$ vs. predicted $\:{T}_{topcoat}$, and (e) actual $\:n$ vs. predicted $\:n$.

In Fig. 9, the prediction results of model based on LSTM + attention trained with only simulation data is plotted. In Fig. 9(a), epochs vs. loss for train and test set is shown. Similar to the 1D-CNN, the LSTM + Attention model quickly stabilizes, reflecting its capability to handle complex dependencies within the simulation data. In Fig. 9(b), 9(c), 9(d) and 9(e) shows the prediction results of $\:k$, $\:\rho\:{C}_{p}$, $\:{T}_{topcoat}$ and $\:n$ respectively, for test dataset. Provides tighter clustering in predictions for all properties compared to the 1D-CNN, particularly for $\:{T}_{topcoat}$ and $\:n$, demonstrating its superior ability to capture temporal and cross-property interactions.

### Training and evaluation with simulation and experiment data

**Fig. 10:**
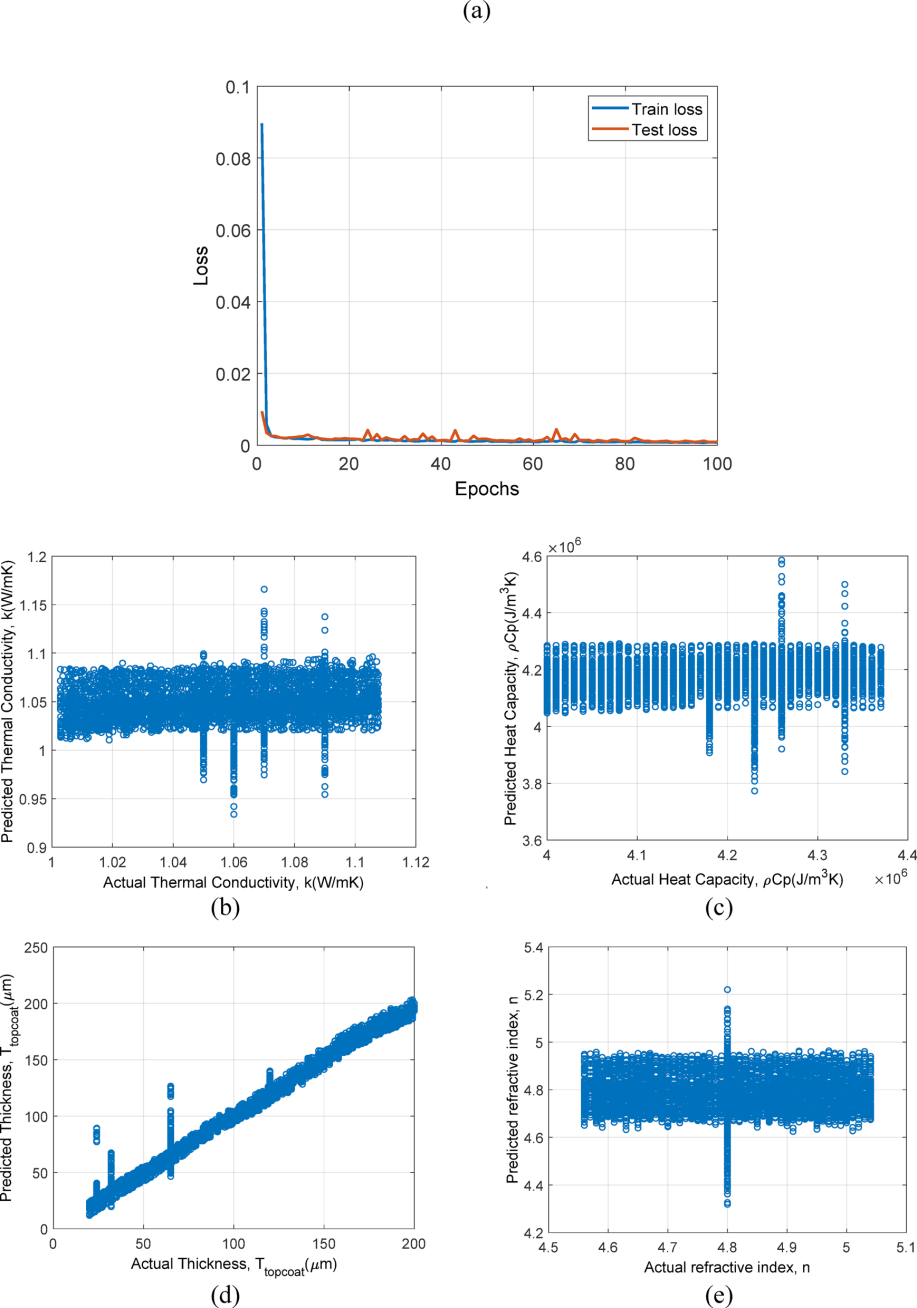
Prediction results for 1D-CNN framework trained and tested on simulation + experiment data: (**a**) epochs vs. loss, (**b**) Actual $\:k$ vs. predicted $\:k$ values, (**c**) actual $\:\rho\:{C}_{p}$ vs. predicted $\:\rho\:{C}_{p}$, (**d**) actual $\:{T}_{topcoat}$ vs. predicted $\:{T}_{topcoat}$, and (e) actual $\:n$ vs. predicted $\:n$.

In this section, models which are trained and tested with only simulation data are discussed. In Fig. 10., the prediction results of model based on 1D-CNN trained with simulation and experiment data is plotted. In Fig. 10(a), epochs vs. loss for train and test set is shown. The rapid convergence and lower final loss values than with simulation data alone, suggesting that the inclusion of experimental data enhances model training and generalization. In Fig. 10(b), 10(c), 10(d) and 10(e) shows the prediction results of $\:k$, $\:\rho\:{C}_{p}$, $\:{T}_{topcoat}$ and $\:n$ respectively, for test dataset. Shows improved accuracy and reduced scatter across all properties, especially $\:k$ and $\:\rho\:{C}_{p}$, indicating that real-world data helps in refining the model predictive capabilities.

**Fig. 11:**
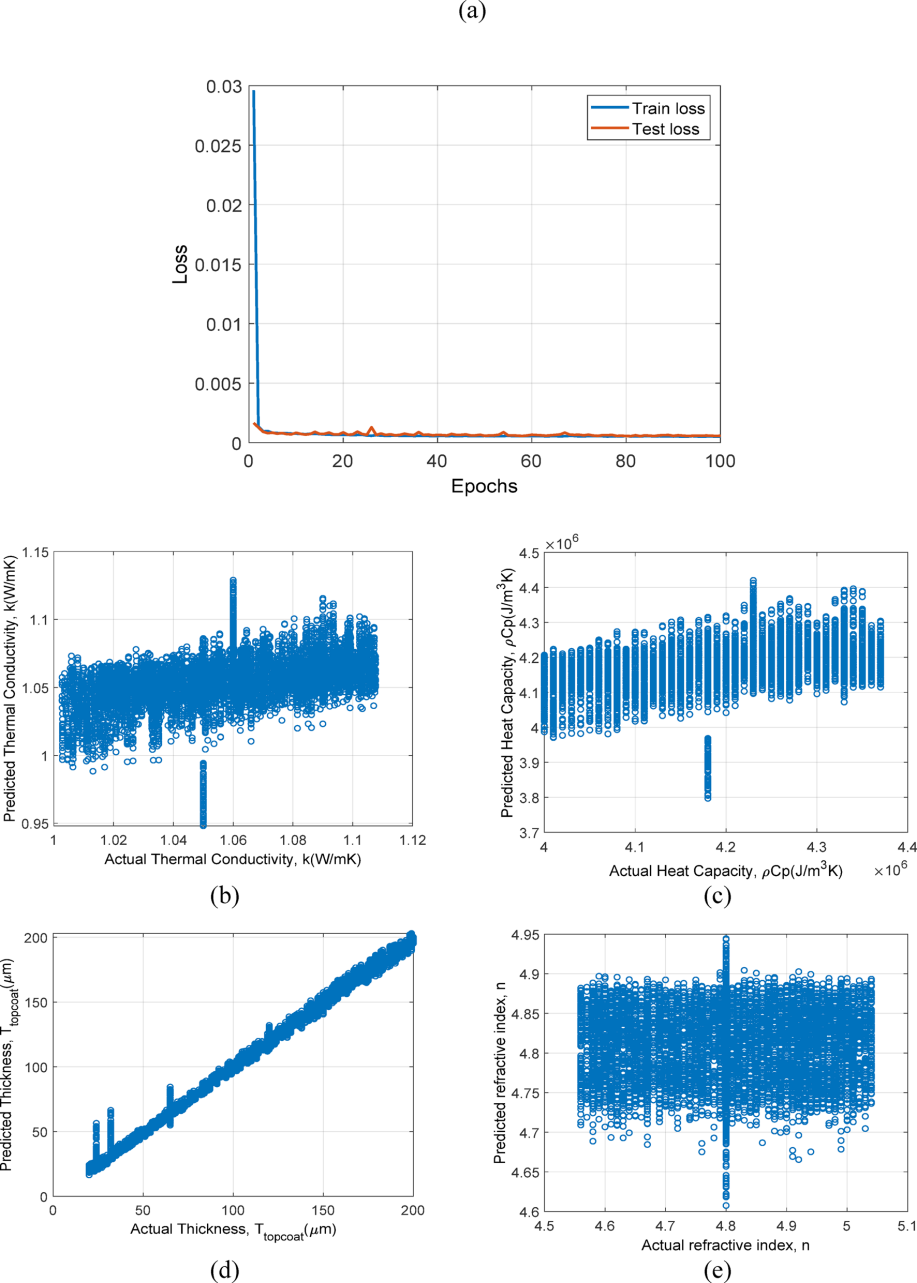
Prediction results for LSTM + Attention framework trained and tested on simulation + experiment data: (**a**) epochs vs. loss, (**b**) Actual $\:k$ vs. predicted $\:k$ values, (**c**) actual $\:\rho\:{C}_{p}$ vs. predicted $\:\rho\:{C}_{p}$, (**d**) actual $\:{T}_{topcoat}$ vs. predicted $\:{T}_{topcoat}$, and (**e**) actual $\:n$ vs. predicted $\:n$.

In Fig. 11., the prediction results of model based on LSTM + Attention trained with simulation and experiment data is plotted. In Fig. 11(a), epochs vs. loss for train and test set is shown. Achieves low and stable losses quickly, demonstrating excellent adaptation to the richer dataset. In Fig. 11(b), 11(c), 11(d) and 11(e) shows the prediction results of $\:k$, $\:\rho\:{C}_{p}$, $\:{T}_{topcoat}$ and $\:n$ respectively, for test dataset. Exhibits the most precise predictions, with very tight clustering and minimal scatter, reinforcing the benefits of combining LSTM’s temporal dynamics handling with diverse training data.

### Prediction results on real-world TBC samples

In this section, the validation of two trained and tested model frameworks on real-world samples with average topcoat thickness ranges from 24 μm to 120 μm is discussed. In Table 1, predictive performance of the 1D-CNN and LSTM with attention models for thermal conductivity and heat capacity under two data training scenarios are compared: simulation-only and simulation combined with experimental data. The key observations from results are: In the case of simulation-only training, the predicted k values with LSTM + Attention outperformed the 1D-CNN with lower MAPE values across all samples. For instance, for Sample 3, LSTM achieved a MAPE of 1.61% compared to 3.18% for CNN. When training is done with simulation and experimental data, both models exhibited improved accuracy. The LSTM model showed increase in prediction performance achieving MAPE values as low as 2.06% for Sample 1 and 4.43% for Sample 4. CNN also showed an increase but maintained higher MAPE values compared to the LSTM with attention. Similar trends were observed for heat capacity predictions. LSTM with attention achieved superior accuracy under both training scenarios, with MAPE values as low as 1.49% (Sample 3) in the simulation-only scenario and 2.05% (Sample 4) in the combined data scenario. The CNN model shows acceptable prediction performance but remained less robust, with MAPE values exceeding 4% for some samples for simulation-only training.

**Table 1:** Prediction results of thermal conductivity ($\:k$) and heat capacity ($\:\rho\:{C}_{p}$) for real-world TBC samples.

Input data	Model	Sample	Thermal conductivity, *k* (W/mK)			Heat capacity, $\:\varvec{\rho\:}{\varvec{C}}_{\varvec{p}}$ (J/m^3^ K) ×10^6^		
			Mean	SD	MAPE	Mean	SD	MAPE
**Simulation**	**1D-CNN**	1	1.04	0.03	4.96	4.19	0.13	3.90
		2	1.02	0.02	3.39	4.12	0.08	2.57
		3	1.05	0.03	3.18	4.25	0.14	2.80
		4	1.02	0.03	3.53	4.14	0.11	2.40
	**LSTM** **+** **Attention**	1	1.07	0.01	2.80	4.27	0.06	2.32
		2	1.08	0.01	3.7	4.31	0.05	3.24
		3	1.06	0.01	1.61	4.22	0.05	1.49
		4	1.01	0.05	4.86	4.04	0.17	4.48
**Simulation** **+** **Experiment**	**1D-CNN**	1	1.02	0.03	6.29	4.14	0.12	4.70
		2	1.01	0.02	4.60	4.09	0.08	3.26
		3	1.02	0.02	4.93	4.09	0.08	3.80
		4	1.05	0.02	2.04	4.22	0.08	1.97
	**LSTM** **+** **Attention**	1	1.06	0.01	2.06	4.26	0.05	2.05
		2	1.08	0.02	2.69	4.27	0.09	1.93
		3	1.05	0.01	1.40	4.18	0.05	1.77
		4	1.01	0.04	4.43	4.08	0.15	3.57

**Table 2:** Prediction results of topcoat thickness measurement ($\:{T}_{topcoat}$) and refractive index ($\:n$) for real-world TBC samples.

Training data	Model	Sample	Thickness, $\:{\varvec{T}}_{\varvec{t}\varvec{o}\varvec{p}\varvec{c}\varvec{o}\varvec{a}\varvec{t}}$ (*µ*m)			Refractive index, $\:\varvec{n}$		
			Mean	SD	MAPE	Mean	SD	MAPE
**Simulation**	**1D-CNN**	1	30.03	5.01	25.13	4.72	0.15	3.06
		2	40.43	4.97	26.35	4.64	0.11	3.31
		3	70.09	3.19	7.84	4.78	0.13	2.59
		4	126.56	2.34	5.47	4.82	0.05	1.00
	**LSTM** **+** **Attention**	1	27.96	3.01	16.5	4.84	0.03	1.12
		2	37.11	2.97	15.96	4.87	0.02	1.47
		3	68.24	2.19	4.98	4.79	0.05	0.90
		4	124.06	1.86	3.38	4.72	0.06	1.67
**Simulation** **+** **Experiment**	**1D-CNN**	1	29.06	3.99	21.08	4.69	0.16	3.28
		2	39.49	3.78	23.40	4.62	0.10	3.67
		3	68.05	2.09	4.69	4.63	0.10	3.49
		4	122.98	1.45	2.48	4.83	0.09	1.78
	**LSTM** **+** **Attention**	1	26.76	2.67	11.53	4.8	0.01	0.27
		2	35.89	2.56	12.16	4.82	0.03	0.70
		3	67.93	2.02	4.50	4.78	0.02	0.51
		4	122.04	1.07	1.75	4.77	0.05	1.05

**Fig. 12:**
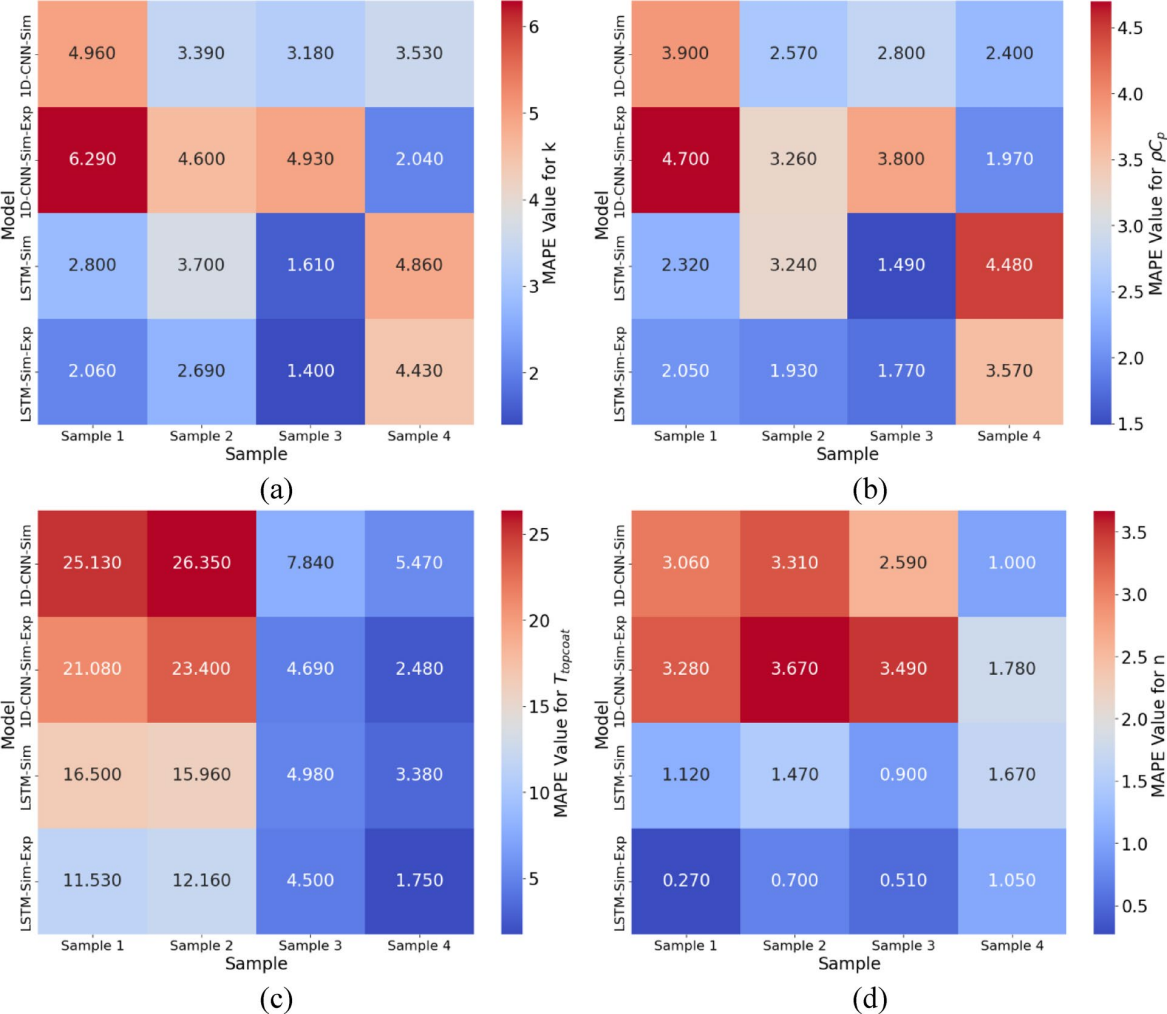
Heatmaps showing the MAPE values for four key predicted properties of TBCs across different models and samples: (**a**) MAPE values for thermal conductivity $\:k$, (**b**) heat capacity $\:\rho\:{C}_{p}$, (**c**) topcoat thickness $\:{T}_{topcoat}$, and (**d**) refractive index $\:n$.

The prediction results for topcoat thickness and refractive index are presented in Table 2, which shows model performances in both data training cases: The LSTM with attention model showed high accuracy in predicting thickness across all samples. It obtained MAPE values as low as 3.38% for Sample 4 with model trained on simulation data, whereas the CNN model obtained higher MAPEs (e.g., 5.47% for Sample 4). The accuracy of both models was significantly improved by the addition of experimental data. The MAPE range of the LSTM model had been 1.75% to 11.53% across all samples, with Sample 4 showing particularly high accuracy of 1.75%. In Fig. 12., shows heatmaps showing the MAPE values for four key predicted properties of TBCs across different models and samples: (a) MAPE values for thermal conductivity $\:k$, (b) heat capacity $\:\rho\:{C}_{p}$, (c) topcoat thickness $\:{T}_{topcoat}$, and (d) refractive index $\:n$. Each row represents a model type, 1D-CNN and LSTM with simulation-only data (Sim) and simulation + experiment data (Sim-Exp). Each column indicates the respective TBC sample used for testing. Lower MAPE values (in blue) indicate better prediction accuracy, while higher values (in red) highlight larger prediction deviations. These visualizations compare the performance of the deep learning models across different input domains and TBC properties. The MAPEs of the CNN model ranged from 2.48% to 21.08% but remained less accurate than LSTM. The LSTM with attention consistently outperformed the CNN, even though both models exhibited high accuracy in predicting the refractive index. For example, in the combined data case, the LSTM achieved a MAPE of 1.05% for Sample 4 and only 0.27% for Sample 1. Even though the CNN model gained advantage from the addition of experimental data, its MAPEs were a little higher than those of the LSTM, particularly for Samples 1 and 2.

Deep learning frameworks are demonstrated to be effective in evaluating properties in the study. During training, both the 1D-CNN and LSTM + Attention models show stable convergence, with a low final loss and fast convergence. Experimental data is added to improve the performance of the model, and the LSTM + Attention model shows higher accuracy and robustness, particularly for thermophysical properties. The predicted and actual $\:k$ values are in line in the 1D-CNN model, and cluster near the diagonal line. However, the model gives slightly higher MAPE values, and scattering increases as k values increase. Higher accuracy is shown by the LSTM + Attention model, which exhibits reduced scatter across all samples. The combination of experiment and simulation data achieves a MAPE as low as 2.06%, further improving prediction performance.

The 1D-CNN predictions align reasonably well with actual $\:\rho\:{C}_{p}$ values but show a broader spread, particularly in the upper and lower bounds of the dataset. The LSTM + Attention model consistently outperforms the 1D-CNN, achieving MAPE values as low as 1.49% with simulation data and 2.05% with combined data. Both models show strong performance for $\:{T}_{topcoat}$ and $\:n$, showing their applicability for NDE in industrial applications. The LSTM with attention model consistently outperformed the CNN across all evaluation measures in both training conditions. The LSTM has the ability to capture temporal dependencies and important features through the attention layer improved its performance. The addition of experimental data to the simulation data significantly improved model accuracy for both predicting thermophysical properties. This highlights the importance of utilizing varied data types for effective deep learning model training. The lowest MAPE values obtained by the LSTM with attention model indicate its fit for accurate evaluation of TBCs. The proposed framework provides an efficient tool for the non-destructive assessment of multiple coating parameters by integrating data from IRT and THz data. In conclusion, the study underscores the potential of multimodal data fusion and deep learning techniques to advance application of NDT in industries.

## Conclusion

This study demonstrates the potential of simulation-assisted multimodal deep learning framework for the evaluation of TBCs using IR and THz inspection data. Mainly two deep learning frameworks are developed based on 1D-CNN and LSTM based on attention. These models are compared for predicting the key thermophysical and optical properties of TBCs, including $\:k$, $\:\rho\:{C}_{p}$, $\:{T}_{topcoat}$ and $\:n$ simultaneously. The prediction results highlight the effectiveness of multimodal data fusion and role of combining experiment and simulation data for training DL models to improve the performance.

**Key outputs of this study**:

•The LSTM + Attention model consistently showed higher prediction accuracy compared to 1D-CNN model in predicting all properties, achieving lower MAPE values and demonstrated efficiency in learning features from complex temporal profiles of IRT and THz.
•The addition of experiment data combined with simulation data in training DL models significantly improved the prediction accuracy of properties, which lead the way for the building of more generalized models.
•1D-CNN and LSTM + Attention models showcased excellent prediction accuracy for $\:{T}_{topcoat}$ and $\:n$ with MAPE as low as 1.75% and 0.27%, respectively, when using model trained with combined data. However, LSTM + Attention model exhibits higher prediction accuracies for $\:k$ and $\:\rho\:{C}_{p}$ with lesser MAPE values for all four samples.

The proposed frameworks provide a more efficient and accurate method for the AI integrated NDE of TBCs in industrial applications, such as energy production and aerospace components. The ability to integrate data from IRT and THz imaging enables characterization of TBCs, by overcoming the limitations of existing single-modality NDE inspection techniques. While existing studies using either IRT or THz data have focused mainly on estimation of thickness or porosity, our model simultaneously estimates multiple properties including heat capacity and refractive index with high accuracy.

The generalizability and prediction accuracy of AI models makes them suitable for applications such as predictive maintenance and lifetime estimation of high-performance coatings. The proposed study has limitations due to limited availability of samples with different service conditions and material composition for collecting experiment data. Also, in both IRT and THz simulations included approximations of models which might have contributed the error in the parameter predictions.

The experimental validation is limited to only four samples and current results primarily showcase the proof-of-concept performance. However, the large dataset based on physics-based simulation helps in improving generalization and mitigates overfitting. The dataset will be extended to include samples with wide range of degradation, variable bond-coat thicknesses, material compositions and service conditions in the future study. Additionally, the reliability and scalability of the proposed frameworks for greater industrial applications can be improved further by incorporating advanced simulation techniques and investigating alternative machine learning architectures. In summary, this research provides a strong basis for the application of deep learning and multimodal data integration to the advancement of TBC evaluation, thereby facilitating the development of automated NDE methods that are more efficient and reliable in critical industrial environments that are focused on NDE 4.0.

## Data Availability

The data that support the findings of this study are available upon reasonable request from the corresponding author, Sruthi Krishna Kunji Purayil.

## Code Availability

The code for the Sim-MDL models mentioned in the paper is available at https://github.com/SKrishkp/Sim-MDL.
